# Experimental Design and Optimization of Nano-Transfersomal Gel to Enhance the Hypoglycemic Activity of Silymarin

**DOI:** 10.3390/polym14030508

**Published:** 2022-01-27

**Authors:** Marwa H. Abdallah, Amr S. Abu Lila, Seham Mohammed Shawky, Khaled Almansour, Farhan Alshammari, El-Sayed Khafagy, Tarek Saad Makram

**Affiliations:** 1 Department of Pharmaceutics, College of Pharmacy, University of Ha’il, Ha’il 81442, Saudi Arabia; kh.almansour@uoh.edu.sa (K.A.); frh.alshammari@uoh.edu.sa (F.A.); 2 Department of Pharmaceutics and Industrial Pharmacy, Faculty of Pharmacy, Zagazig University, Zagazig 44519, Egypt; a.abulila@uoh.edu.sa; 3 Department of Pharmaceutics and Pharmaceutical Technology, Faculty of Pharmacy, Al-Azhar University, Cairo 11651, Egypt; sehamshawky@azhar.edu.eg; 4 Department of Pharmaceutics, College of Pharmacy, Prince Sattam Bin Abdulaziz University, Al-Kharj 11942, Saudi Arabia; e.khafagy@psau.edu.sa; 5 Department of Pharmaceutics and Industrial Pharmacy, Faculty of Pharmacy, Suez Canal University, Ismailia 41552, Egypt; 6 Department of Pharmaceutics and Industrial Pharmacy, Faculty of Pharmacy, October 6 University, Al Mehwar Al Markazi 12511, Egypt; tareksaadmakram@yahoo.com

**Keywords:** Silymarin, transfersomes, Box Behnken Design, hypoglycemic effect, transdermal application

## Abstract

Current advancements in the research investigations focused at using natural products to generate novel dosage forms with a potential therapeutic impact. Silymarin is a natural product obtained from the herb *Silybum marianum* that has been shown to have remarkable hypoglycemic activity. Owing to the low enteral absorption, instability in stomach secretion, and poor solubility of Silymarin, it was better to be produced as a topical dosage form. A three-factor, three-level Box Behnken (3^3^ BB) design was constructed to develop 15 formulations using three independent variables (phospholipid concentration, surfactant concentration, and sonication time) and two dependent variables (encapsulation efficiency and in vitro drug release). The optimized formula was added to HPMC gel and the resulting transfersomal gel was investigated for its characteristics, in vitro, ex vivo and hypoglycemic behaviors. The pH of the Silymarin-loaded transfersomal gel was 7.05, the spreadability was 55.35 mm, and the viscosity was 6.27 Pa. Furthermore, Silymarin loaded transfersomal gel had the greatest transdermal flux (92.41 µg/cm^2^·h), which was much greater than all other formulations. In vivo observations revealed that Silymarin loaded transfersomal gel significantly reduced blood glucose levels, compared to either Silymarin gel or oral Silymarin suspension. The findings show that the developed transfersomal gel could be an effective carrier for Silymarin transdermal delivery.

## 1. Introduction

Transdermal drug delivery systems (TDDS) have been exploited for many years to deliver drugs [1]. The transdermal bioactive agent has to pass through skin layers to reach the systemic circulation. The penetrated drug is then transported via the blood stream to the whole body to exert its pharmacological action. Compared to other routes of administration, the transdermal route of administration exhibits potential benefits, such as evading first pass hepatic metabolism, extending drug duration of action, minimizing adverse effects, enhancing the pharmacological action, minimizing the fluctuation in drug concentrations, and improving patient’s convenience [2]. Most notably, TDDS can be effectively implemented when drug therapy is essential for chronic uses or for a prolonged time. Therefore, the development of TDDS for treating a variety of pathological conditions, such as diabetes, is a viable option. Nevertheless, transdermal therapy is restricted to certain types of bioactive agents, as the stratum corneum poses a barrier against the permeating substances [3].

The use of nano-formulations has emerged as a viable mean to circumvent limitations associated with transdermal therapy [4]. Due to the merits of small particle size, better drug retention, along with their targeting ability, nano-formulations have been considered ideal TDDSs. Accordingly, many approaches have been adopted to enhance the transdermal delivery of bioactive agents using nanoparticulate drug delivery systems, such as liposomes [5], transfersomes, ethosomes [6], dendrimers and microemulsions [7]. Liposomes as one of the transdermal delivery systems have been studied since the 1980s and have attracted a lot of interest. Nevertheless, liposomes do not penetrate deeply into the skin of rats and are confined to the upper layer of the skin [8]. By contrast, transfersomes, ultra-flexible liposomes, represent a promising lipid-based vesicular system that is extensively exploited in the field of transdermal drug delivery [9]. As a result of their ultra-flexible membrane characters, they have the ability to deliver the drug either into or through the skin, depending on the application, with high efficacy [10]. The vesicular transfersomes are more elastic than other vesicular delivery systems, such as liposomes, and thus well suitable for the skin penetration [11].

Recently, there has been a surge in interest in using herbal medicines for the treatment of various disease. Silymarin is a natural polyphenolic flavonoid extracted from milk thistle seeds (*Silybum marianum* L.); Silibinin (Silybin) is its main bioactive ingredient. Silymarin is a well-known hepatoprotective medication that has been proven in numerous in vitro and in vivo animal models to exhibit antioxidant [12], anti-inflammatory/immunomodulatory [13], and antifibrotic activities [14]. Several animal model studies have recently suggested that Silymarin may have potential anti-diabetic and lipid-lowering characteristics [15,16].

Response surface methodology (RSM) investigates the impact of a number of explanatory factors on one or more response variables. Generally, an experimental design entails selecting the proper combination of independent factors and the level of each factor to be investigated. Nevertheless, because experimental runs are costly in terms of both time and money, it is important to keep the number of runs to a minimum while still achieving the required results. To achieve this, some techniques such as Box–Behnken (BB) [17], full factorial, central composite designs [5] are widely employed. Optimization with factorial designs and response surface analysis are effective approaches for minimizing the time required for the development of pharmaceutical dosage forms and improving research output [17].

The current investigation is focusing on developing an effective delivery vehicle for natural products like Silymarin. Our goal was to (i) develop Silymarin loaded transfersomes (SmTFs), which were then optimized using a 3^3^ Box–Behnken Design (BBD). The optimized Silymarin loaded transfersomes were incorporated into HPMC to prepare transfersomal gel loaded with Silymarin. Subsequently, the skin permeability properties of the developed transfersomal gel were studied; (ii) investigate whether treatment with the newly formulated Silymarin loaded transfersomal gel can improve the capability of Silymarin to reduce the elevated blood glucose level. To the best of our knowledge, no previous research has attempted into the use of transfersomal gel as a delivery system for the transdermal delivery of Silymarin.

## 2. Materials and Methods

### 2.1. Materials

Silymarin (SM) was a gift sample obtained from Sigma Pharmaceutical Industries (Nasr City, Cairo, Egypt). Phospholipone H 100 (Pl), Span 80 and sodium azide were procured from Sigma Chemical Co. (St. Louis, MO, USA). Tween 80, chloroform and methanol were purchased from El-Nasr Pharmaceutical Chemical Co. (Cairo, Egypt). HPMC was provided from El-Nile pharmaceutical company (Cairo, Egypt).

### 2.2. Statistical Modelling for Optimizing the Silymarin-Loaded Transfersomes Formulation

Optimization of the formulated Silymarin loaded transfersomes was performed using Box–Behnken Design (BBD) as one of the response surface methodology (RSM) tools. Basically, three factors three levels (3^3^) Box–Behnken Design was constructed using three independent variables representing phospholipid concentration (X_1_), surfactant concentration (edge activator, EA) (X_2_) and sonication time (X_3_), with three levels being high (+1), medium (0) and low (−1) as demonstrated in Table 1.

The dependent variables examined were encapsulation efficiency, EE% (Y_1_), and in vitro release of the drug after 6 h (Y_2_). The Design-Expert version 12.0 software (Stat-Ease, Minneapolis, MN, USA) was used for evaluation of the effects of formulation variables on the investigated dependent variables. Fifteen runs were prepared according to the experimental design to obtain the optimized formula with the desired responses. Analysis of variance (ANOVA) test was adopted to analyze the obtained data for assessing the model significance and prove the statistical analysis of the data. In order to assess the formulation responses, a statistical model introducing interactive and polynomial terms was employed given by equation below:

Y = b_o_ + b_1_X_1_ + b_2_X_2_ + b_3_X_3_ + b_12_X_1_X_2_ + b_13_X_1_X_3_ + b_23_X_2_X_3_ + b_11_X_1_^2^ + b_22_X_2_^2^ + b_33_X_3_^2^ (1)

where Y indicates the dependent response while b_0_ symbolizes the intercept; b_1_, b_2_, b_3_, b_12_, b_13_, b_23_, b_11_, b_22_ and b_33_ denote the regression coefficients. X_1_, X_2_ and X_3_ represent the main factors, X_1_X_2_, X_1_X_3_, X_2_X_3_ indicate the interactions between main factors and X_1_^2^, X_2_^2^, X_3_^2^ represent the polynomial terms. The p-values related to the regression coefficients indicated the significance of the independent factors on the dependent responses.

### 2.3. Preparation of Silymarin Loaded Transferosomes

Transfersomal formulations were developed by Rotary Flask Evaporation Sonication technique previously described by Abdallah [18] using Box–Behnken model. Precise amounts of phospholipids, Sorbitan mono-oleate (Span 80; edge activator), and Silymarin were dissolved in a mixture of methanol and chloroform (1:1, *v*/*v*). The organic solvents mixture was slowly evaporated at 60 °C under reduced pressure using the rotary evaporator (Buchi rotavapor R-3000, Flawil, Switzerland). The formed dried thin lipid film was subjected to hydration using 10 mL phosphate buffer solution (PBS; pH 7.4), while keeping mild agitation in water bath at 60 °C for one hour to get transfersomal dispersion. The transfersomes were left for additional 2 h at room temperature for swelling. Subsequently, transfersomal vesicles were sonicated for 20–30 min using bath sonicator (Model Julabo Labortechnik GMBH, Seelbach, Germany). Fifteen formulations were prepared according to the experimental design, the encapsulation efficiency (EE%), and in vitro release after 6 h of Silymarin transfersomes (SmTFs) are presented in Table 2.

### 2.4. Characterization of Silymarin Loaded Transfersomes (SmTFs)

#### 2.4.1. Determination of Vesicle Size

Vesicle size of the optimized transfersomal vesicles was carried out using Zetasizer apparatus (Mastersizer 2000 version 5.22, Malvern Instruments Ltd., Worcestershire, UK). The dynamic light scattering technique was utilized for assessing the particle size of the formulations [5,19].

#### 2.4.2. Encapsulation Efficiency Determination (EE%)

The encapsulation efficiency of transfersomal dispersions loaded with Silymarin (SmTFs) was determined by centrifuging the dispersion at 6.000 rpm for 60 min at 4 °C [6]. After centrifugation, the supernatant was taken and diluted, then the absorbance was monitored at λ_max_ 287 nm using spectrophotometer (Shimadzu UV/VIS, Tokyo, Japan) [20]. The percentage encapsulation efficiency was calculated from the following equation [21]:

% Drug Encapsulation efficiency = (A_T_ − A_F_)/A_T_ × 100 (2)

where, A_T_ is the total amount of Silymarin in transfersomal dispersions and A_F_ is the free amount of Silymarin that was found in the supernatants.

#### 2.4.3. In Vitro Drug Release from Different Transfersomal Preparations

In vitro drug release study was performed to determine the percentage of Silymarin released from the fabricated transfersomal formulations. The in vitro release investigation of drug was inspected according to technique previously described by Ibrahim et al., with minor modification [22]. Briefly, the in vitro drug release through cellophane membrane (MW cut-off 12,000–14,000 Da), which only allows the diffusion of free drug while it retains lipid vesicles, was performed using locally fabricated diffusion cells (Figure S1). Transfersomal dispersions were put in glass tubes that were closed on one side with a dialysis membrane, that had been presoaked in the release medium, and secured with a rubber band. These tubes were immersed in 250 mL phosphate buffer, pH 7.4, maintained at 37 ± 0.5 °C and set at a rotational speed of 50 rpm. At predetermined time points (0.25, 0.5, 1, 2, 4 and 6 h), 2 mL samples were withdrawn and analyzed spectroscopically at λ_max_ 287 nm. The samples were replaced with the same volume of fresh buffer.

### 2.5. Stability Studies of the Optimized Transfersomal Formulation (SmTFs)

The stability of the optimized transfersomes loaded with Silymarin (SmTFs) was investigated based on measuring some parameters such as vesicle size, entrapment efficiency percentage and percentage of in vitro drug release after 6 h. The study was conducted in accordance with the guidelines of ICH. Samples from the optimized transfersomal formulation were stored in tightly closed containers and kept at two different conditions: 4 ± 1 °C and at 25 ± 1 °C for 1 and 3 months [21].

### 2.6. Prepatation of Silymarin Loaded Gel

#### 2.6.1. Formulation of Silymarin Gel

Silymarin gels 1% *w*/*w* were formulated using HPMC (4%) as gelling agents [23,24]. Four grams of HPMC was gently sprinkled on hundred milliliters of phosphate buffer saline containing Silymarin and rotated at 400 rpm using magnetic stirrer (Heating Magnetic Stirrer–AREC, VELP Scientifica, Milano, Italy) until a thin homogenous dispersion was achieved [25].

#### 2.6.2. Formulation of Silymarin Transfersomal Gel

Silymarin-loaded transfersomal gels were manufactured by substituting a part of the PBS with a concentrated transfersomal dispersion containing the required quantity of drug and performing the technique as described earlier.

### 2.7. Evaluation of the Prepared Silymarin Loaded Transfersomal Gel

#### 2.7.1. Physical Inspection

The developed gel formulations loaded with Silymarin was inspected visually to assess the homogeneity of the formulations.

#### 2.7.2. Estimation of pH Value

The pH measurement of Silymarin transfersomal gels was investigated using a calibrated digital pH meter (Jenway 3510, Fisher Scientific UK Ltd., Loughborough, UK) at room temperature [26]. The pH measurement was triplicated and the average reading was taken.

#### 2.7.3. Spreadability Test

The purpose of this experiment was to investigate the spreadability of the developed gel and measure the diameters of spreading when applied to the affected area. Briefly, gel was retained between two slides and a definite weight was fastened for 1 min over the upper slide. The spreading area diameter was measured as an indication of the spreadability [7,27].

#### 2.7.4. Rheological Studies and Viscosity

Viscostar-R rotational viscometer (Fungilab S.A., Barcelona, Spain) was used to measure the viscosity of the developed transfersomal gels at 25 °C using Spindle R5 at 2 rpm. The viscosity determination was carried out in triplicate and the mean reading was taken [8,23].

#### 2.7.5. Drug Content Determination

Accurately, an amount of 0.5 g of the developed gel preparations (equivalent to 5 mg of Silymarin) was diluted to ten milliliters using phosphate buffer saline, pH 7.4. The drug content was spectrophotometrically analyzed at λ_max_ 287 nm using a blank sample containing the same components (without drug). The percentage of drug content was calculated as follow:

(3) $$\%\;\mathrm{Drug}\;\mathrm{content}=\frac{\mathrm{Actual}\;\mathrm{amount}\;\mathrm{of}\;\mathrm{the}\;\mathrm{drug}\;\mathrm{in}\;\mathrm{the}\;\mathrm{formulation}}{\mathrm{Theoretical}\;\mathrm{amount}\;\mathrm{of}\;\mathrm{the}\;\mathrm{drug}\;\mathrm{in}\;\mathrm{the}\;\mathrm{formulation}}\times 100$$

### 2.8. In Vitro Drug Release from Transfersomal Gel

As discussed previously in Section 2.4.3, the same methodology was used in order to assess the release rate of Silymarin from the developed transfersomal gel formulations, compared to free drug, transfersomes and Silymarin gel preparation. At definite time intervals (0.25, 0.5, 1, 2, 4 and 6 h), 2 mL samples were withdrawn and substituted with the fresh buffer. Samples were analyzed for drug content spectroscopically at λ_max_ 287 nm [28].

### 2.9. Ex Vivo Drug Permeation Study

White Albino male rabbits’ abdominal full-thickness skins (1–1.2 mm thickness) were used. For the experiment, the skin of the animals was carefully removed and processed. The skin samples that had been produced were placed on the receptor compartment. The dermis was pointing downward to the media, whereas the stratum corneum was pointing upward to the sample. After that, 250 mL of PBS with 0.02 percent sodium azide, as a preservative, kept at 37 ± 0.5 °C was used, representing the receptor media. The experiment was performed as previously stated in Section 2.8. Briefly, one gram of each formulation equivalent to 10 mg of Silymarin were put in glass tubes that were closed on one side with skin samples (the stratum corneum side with diameter of 2.8 cm and surface area of 6.15 cm^2^) and secured with a rubber band. These tubes were immersed in 250 mL phosphate buffer, pH 7.4, with 0.02 percent sodium azide, as a preservative, maintained at 37 ± 0.5 °C and set at a rotational speed of 50 rpm. At predetermined time points (0.25, 0.5, 1, 2, 4 and 6 h), 2 mL samples were withdrawn and analyzed spectroscopically at λ_max_ 287 nm. The samples were replaced with the same volume of fresh buffer.

Steady state transdermal flux (J_ss_) and enhancement ratio (ER) were calculated using the following equations:

J_ss_ = Amount of permeated drug/(area of permeation × time);
ER = J_ss_ from test/J_ss_ from control. (4)

### 2.10. In Vivo Experimental Studies

#### 2.10.1. Selection of Animals and Experimental Induction of Diabetes

Male albino-wistar rats weighing 200–250 g were used in this investigation. All tests were carried out in accordance with the recommendations and regulations of Research Ethics Committee (REC) of Ha’il University (20455/5/42). The rats were acclimatized in standardized conditions of temperature and lightening. One hour before the study, the animals were habituated to the laboratory environment. The levels of blood glucose (BGL) for all animals were monitored [11].

Diabetes was induced using the Panda et al. technique. Briefly, the rats were i.p. injected with 120 mg/kg of alloxan monohydrate, freshly dissolved in saline [13]. To overcome the hypoglycemia, the animals were given 5% glucose solution overnight. Rats showing glucose levels ≥200 mg/dL were classified as diabetic and employed in the experiment [12].

#### 2.10.2. Determination of Blood Glucose Concentration

A day before the experiment, part of skin on the rat’s dorsal side was carefully shaved, and then cleaned with distilled water. The animals were fasted overnight before the studies and were randomized into five groups (*n* = 5). The first group (control group) includes diabetic rats treated with 2 mL of normal saline. The second group (placebo group) includes rats treated with drug-free HPMC gel. The third group comprises diabetic rats treated orally with aqueous Silymarin suspension (50 mg/kg) [24]. The fourth group includes diabetic rats transdermally treated with Silymarin-loaded HPMC gel (1% *w*/*w*). The last group comprises diabetic rats transdermally treated with Silymarin-loaded transfersomal gel (1% *w*/*w*, 50 mg/kg). At definite time intervals (0, 1, 2, 3, 4, 6, and 8 h) post-treatment, one hundred microliters of blood were withdrawn from the tail vein for the detection of glucose. Blood glucose level (BGL) was estimated using a blood glucose monitoring device (One Touch, Lifescan Inc., Milpitas, CA, USA). The percentage lowering in blood glucose level was estimated [29].

### 2.11. Statistical Analysis

The findings were recorded as mean ± standard deviation. The significance of the data was assesed by conducting ANOVA test using SPSS statistics software (Version 14, IBM Corporation, Armonk, NY, USA). Statistical significance is interpreted if *p* is less than 0.05 [30].

## 3. Results and Discussion

### 3.1. Preliminary Studies for Preparation of Silymarin-Loaded Transfersomes (SmTFs)

Transfersomes loaded with Silymarin were prepared using a conventional rotary evaporative sonication technique. This process was chosen because the formation of thin films occurs over a surface area sufficient for full vesicles hydration resulting in an enhancement of the encapsulation efficiency percentage [31]. Preliminary trials were performed to select the surfactant (EA) that produces transfersomes with the highest encapsulation efficacy percentage. It was noticed that the transfersomal formulation prepared using Span 80 as an edge activator (EA) exhibited the highest EE% compared to that prepared from Tween 80 (data not shown). These findings are correlated to the edge activators’ HLB values. Edge activators with a low HLB value (HLB value of Span 80 = 4.3) produce transfersomes with a high EE%, which results from the increased ratio of lipid volume in the transfersomal vesicles to the encapsulated aqueous volume [18]. Consequently, in this study, span 80 was adopted as an EA to give flexibility to the transfersomes’ membrane.

Next, for the formulation and optimization of Silymarin-loaded transfersomes, a three-factor, 3-level Box–Behnken experimental design was adopted. A total of 15 formulations with different amounts of phospholipid, amounts of surfactant, and sonication times were prepared, as shown in Table 2. The amount of phospholipid (X_1_), amount of surfactant (X_2_), and sonication time (X_3_) were set in the range of 100–400 mg, 10–50 mg, and 20–30 min, respectively, as independent factors.

### 3.2. Expermintal Design

#### 3.2.1. Analysis of Box–Behenken Design (BBD)

The relationships between independent variables, such as the amount of phospholipid (X_1_), amount of surfactant (X_2_), and sonication time (X_3_), at three levels (−1, 0, +1), with dependent responses, such as the encapsulation efficiency percentage (Y_1_), and the percentage of the in vitro drug released after 6 h (Y_2_) were assessed by the Box–Behenken Design, using the Design Expert^®^ software (Minneapolis, MN, USA). The quadratic model was found as the optimum model for all two of the dependent responses.

According to the 3^3^ Box–Behnken Design investigations, the amounts of lipid forming vesicles (phospholipid), edge activator concentration (Span 80) and sonication time had a significant impact on the encapsulation efficiency and percentage of the in vitro drug release. These observations ensure the selection of independent variables in this investigation. The significance of the model was estimated by ANOVA, where, at *p*-value < 0.05, the model is considered significant. The *p*-value < 0.05 clarifies that, the quadratic model is statistically significant to describe the interrelationship among the independent factors and the dependent responses.

#### 3.2.2. Effect of Formulation Parameters on the Encapsulation Efficiency (Y_1_)

**Figure 1:**
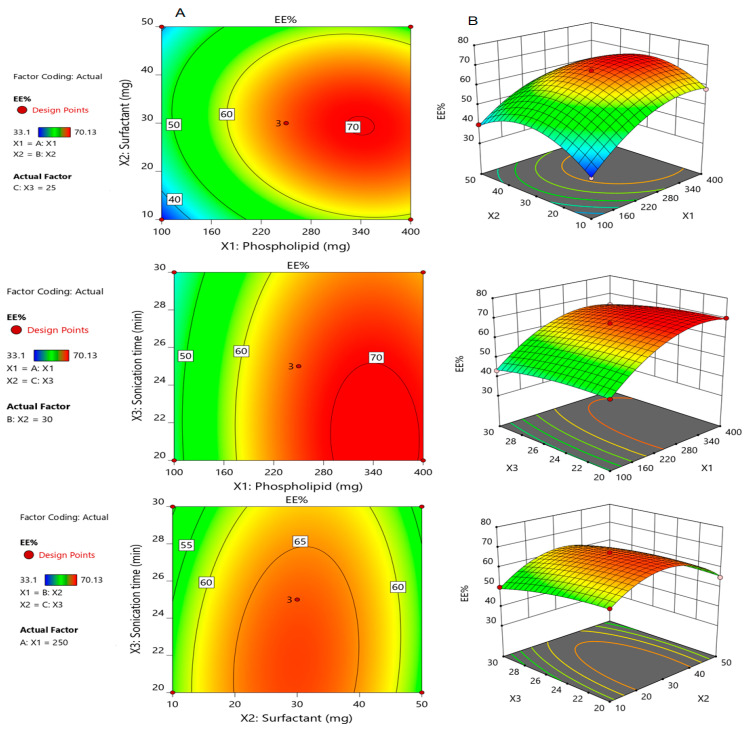
Contour plots (**A**) and corresponding response surface plots (**B**) which show effects of the independent variables on encapsulation efficiency (Y_1_). Two independent variables are considered at a time, while the third one remains constant.

The influences of the independent factors on encapsulation efficiency (EE%) of Silymarin loaded transfersomes (SmTFs) are represented by contour plots and their corresponding 3D response surface graphs. As shown in Figure 1, increasing the Span 80 concentration from 10 mg to 30 mg resulted in a proportional increase in the encapsulation efficiency of the drug. However, a further increment of Span 80 to 50 mg, resulted in a marked decrease in the encapsulation efficiency. As illustrated in Table 2, the maximum entrapment efficiency was found 70.13 ± 0.80% for F9, while the minimum value was 33.10 ± 0.66% for F15.

The incorporation of the low concentration of EA resulted in an increase in the vesicle size, whereas a further increment in the edge activator concentration might trigger pores formations in the bilayers, which become leaky to the encapsulated drug [32]. Moreover, increasing the concentration of EA resulted in the formation of mixed micelles which coexisted with the prepared transfersomes [10]. Similar results were reported by Abdallah who demonstrated that the encapsulation efficiency of Nystatin decreased with the increasing edge activator concentration [18]. In addition, increasing the sonication time resulted in a noticeable reduction in the EE%, which is attributed to the reduction of vesicle size by increasing the sonication time [33].

The fitted mathematical polynomial equation derived from the BBD verified our findings as it demonstrates the synergistic effect of X_1_ and X_2_, and the antagonistic effect of X_3_ on the dependent response Y_1_. A positive coefficient implies that the factor has a synergistic influence, whereas a negative value shows an antagonistic influence on the responses.

Y_1_ = 66.96 + 10.54X_1_ + 0.71X_2_ − 2.48X_3_ −2.17X_1_X_2_ − 0.4X_1_X_3_ − 1.30X_2_X_3_ − 8.85X_1_^2^ −11.31X_2_^2^ − 1.81X_3_^2^ (5)

#### 3.2.3. Effect of Independent Factors on Percentage of Drug Released after 5 h (Y_2_)

In vitro release studies of Silymarin from Silymarin-loaded transfersomes were studied at different time points until 6 h. It was noticed that F11, with a high amount of phospholipid and a medium amount of Span 80, showed maximum drug release after six hours (60.01 ± 0.59%), whereas the minimum drug release was from F15 (28.35 ± 0.28%), with a low concentration of lipid forming vesicles, as summarized in Table 2.

The drug release from transfersomes after 6 h was increased by an increasing amount of Span 80, from 10 to 30 mg at the same concentration of phospholipid. The percentage of drug released for F3, which was composed of 400 mg phospholipid and 10 mg surfactant (51.81 ± 1.22), was less than the percentage of drug released for F9, composed of 400 mg phospholipid and 30 mg surfactant (58.13 ± 1.56). A further increase in the amount of surfactant to 50 mg resulted in a noticeable decrease in the percentage of drug released (50.03 ± 1.13; F12). The lower drug release observed at low Span 80 concentrations could be attributed to the more organized and less leaky lipid membranes of transfersomal vesicles which hindered drug release [18,34]. Additionally, at a high level of surfactant concentration (50 mg), the percentage of drug released was minimal, due to formation of rigid mixed micelles which coexisted with the transfersomal vesicles. Another explanation that supports our finding is that at a high concentration of edge activator, the drug encapsulation efficiency decreased and led to the disruption of the vesicles lipid membranes, which become less ordered and more leaky resulting in the leakage of the encapsulated drug, as described by Mahmood et al. [32].

**Figure 2:**
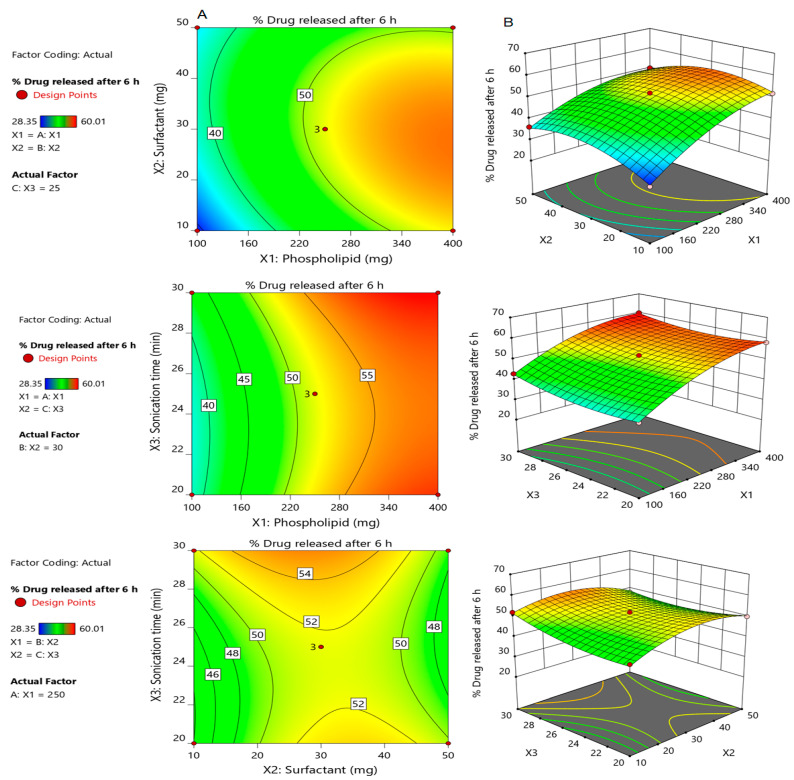
Contour plots (**A**) and corresponding response surface plots (**B**) which show effects of the independent variables on percentage drug released after 6h (Y_2_). Two independent variables are considered at a time, while the third one remains constant.

Our findings were evidenced by the contour plots and their corresponding 3D response surface graphs, Figure 2, which describe the impact of formulation variables X_1_, X_2_ and X_3_ on Y_2_. Furthermore, the interrelationship between the independent factors and their studied dependent variables could be affirmed by the mathematical polynomial equation derived from the BBD and illustrated below:

Y_2_ = 51.65 + 9.30X_1_ + 0.88X_2_ + 1.49X_3_−2.39X_1_X_2_ − 0.78X_1_X_3_ −2.27X_2_X_3_ − 4.48X_1_^2^ − 5.59X_2_^2^ + 2.63X_3_^2^ (6)

#### 3.2.4. Selection of the Optimized Formulation of Silymarin Loaded Transfersomes (SmTFs)

After the construction of the Box–Behnken experimental design, the optimized formulation with the desired properties was specified utilizing the Design Expert^®^ software (Minneapolis, MN, USA) (the point prediction method). The optimized formula was selected from 15 experiments by shifting the criteria towards maximum values of (Y_1_) the encapsulation efficiency percentage, and (Y_2_) the percentage of drug released after 6 h. It was found that the transfersomal formulation, composed of 389.69 mg phospholipid, 30.12 mg Span 80, and at 20 min as sonication time, fits well with prerequisites of an optimum formulation. The optimized formulation showed a 68.61 ± 2.36% entrapment efficiency and a 57.33 ± 2.07% drug release after 6 h, as shown in Table 3.

#### 3.2.5. Validation of the Developed Response Surface Methodology (RSM) Model

**Figure 3:**
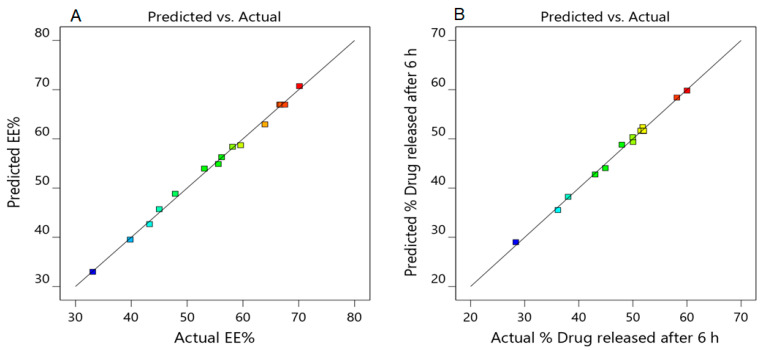
Linear correlation plots between actual and predicted values for all dependent variables; (**A**) for Y_1_; (**B**) for Y_2_.

The theoretical values of both dependent responses Y_1_, and Y_2_, for all developed Silymarin loaded transfersomes, were determined by plugging their corresponding X_1_, X_2_, and X_3_ values into the appropriate mathematical equations generated by the software. The obtained actual and predicted values of the responses were depicted in Table 3. It was obvious that the predicted values and the actual values were in reasonably good agreement as illustrated in Figure 3. These findings confirmed the validity of the developed response Surface Methodology model. Therefore, the generated polynomial equations using BBD could be utilized in predicting the dependent responses values. Figure 3 demonstrates the linear correlation plot of the predicted versus the actual responses indicating that the predicted R^2^ (0.9758) for the Y_1_ response is in reasonable agreement with the adjusted R^2^ (0.9951. Similarly, Figure 3 and Table 4 indicated the linearity of the data through the linear correlation between the adjusted R^2^ value (0.9895) and the predicted one (0.9427) for the Y_2_ dependent response. Additionally, a lack of fit in both the dependents responses Y_1_ and Y_2_ clarified insignificant values (*F*-value), that being 3.58 and 11.23 and *p*-values of 0.2261 and 0.0829 for Y_1_ and Y_3_, respectively (*p* > 0.05), concluding the validity of the model.

#### 3.2.6. Particle Size of the Optimized Silymarin Loaded Transfersomes Formulation

**Figure 4:**
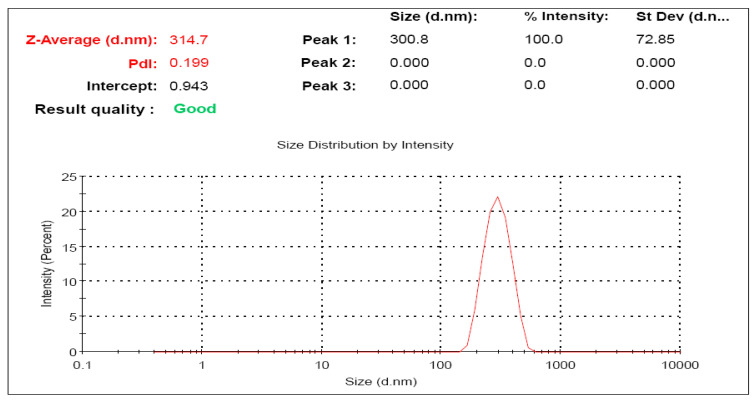
Vesicular size distribution curve of optimized Silymarin loaded transfersomal formulation.

Figure 4 shows the size distribution curve of the optimized Silymarin loaded transfersomes formulation. The average vesicle diameter was 314.7 nm. The size distribution of the vesicles verifies the normal size distribution of the vesicles.

### 3.3. Stability Studies of the Optimized Silymarin Loaded Transfersomes Formulation

**Figure 5:**
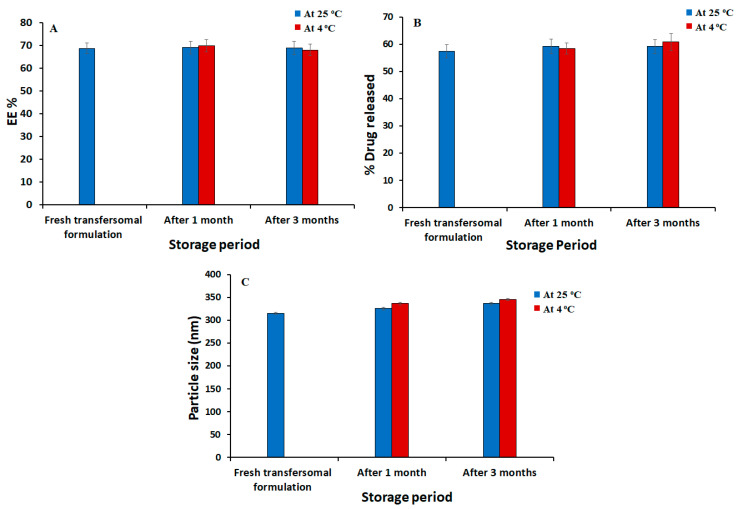
Outline of stability study for optimized Silymarin loaded transfersomal formulation for 1 and 3 months at 4 °C and 25 °C in terms of (**A**) EE%; (**B**) In vitro drug release and (**C**) Particle size (nm) in comparison to freshly prepared formulation.

The results of stability studies for the optimized Silymarin loaded transfersomal preparation (SmTFs) are presented in Figure 5. The obtained results, hours of the preserved transfersomes, did not change significantly over 1 and 3 months at 4 ± 1 °C and 25 ± 1 °C when compared to fresh transfersomes (*p* < 0.05). These findings ascertained the stability and drug-carrying capabilities of transfersomes.

### 3.4. Evaluation of the Developed Silymarin Loaded Transfersomal Gel

Optimized formulation consisting of 389.69 mg phospholipid, 30.12 mg Span 80, and prepared at a sonication time of 20 min was selected for the preparation of the transfersomal gel. The gel was prepared by dispersing the formulation successfully in 4% HPMC and then subjected to further characterization. Transfersomal gel loaded with Silymarin was assessed for a range of parameters, consistency, homogeneity, clarity, pH and spreadability. The gel was found to be smooth, clear, homogeneous and spreadable. The distance traveled by transfersomal gel when compressed between slides was used to estimate spreadability. The gel traveled a total distance of 55.35 ± 3.03 mm. The pH of gel was reported to be 7.05 ± 0.45, which was deemed suitable for skin application [35]. The gel’s viscosity was measured as 6.27 ± 0.63 Pa, indicating that it had enough consistency to be applied to the skin, Table 5.

### 3.5. In Vitro Release Studies

**Figure 6:**
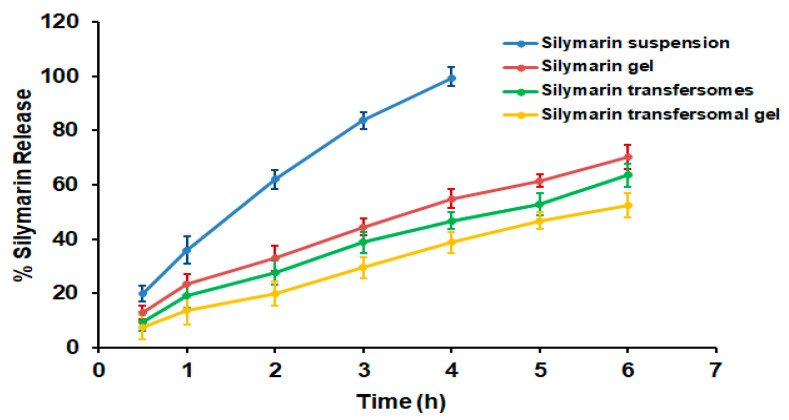
Study of in vitro drug release from different Silymarin formulations and Silymarin suspension at 37 °C. Results are stated as the mean ± SD of three trials.

Figure 6 shows the percentage of Silymarin released from various developed formulations and from drug suspension over the cellophane membrane. The investigation was planned for 6 h. As shown in Figure 6, 99.33 ± 3.25% of Silymarin was released from the Silymarin suspension within the first 4 h, which is a significantly higher than that released from all other prepared formulations (*p* < 0.05). In contrast, after 6 h, 52.55 ± 3.15%, 63.57 ± 2.78% and 70.37 ± 2.56% of Silymarin was released from Silymarin loaded transfersomal gel, Silymarin transfersomes, and Silymarin loaded gel, respectively. Furthermore, the Silymarin gel formulation released a significantly higher percentage of the drug than transfersomes and transfersomal gel (*p* < 0.05), which can be attributed to the highest water content in gel preparation, which speeds up the transfer of Silymarin from the formulation to the release media. It was noticed that transfersomes released significantly more Silymarin than transfersomal gel, which could be due to the decreased viscosity of transfersomes compared to transfersomal gel, which facilitates the flow of the encapsulated drug to the medium.

### 3.6. Ex Vivo Skin Permeation Investigation of Silymarin from Different Formulations

**Figure 7:**
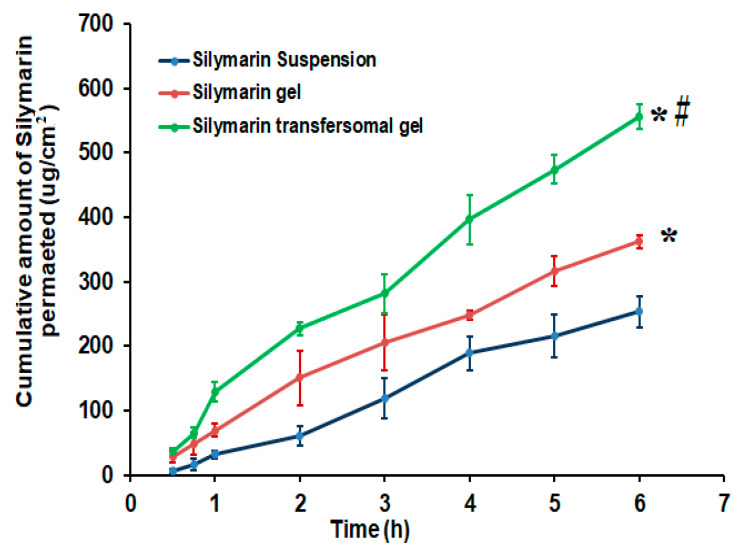
Permeation study of Silymarin from different formulations through excised rat skin compared to Silymarin suspension. Results are expressed as mean ± SD (*n* = 3). * (*p* < 0.05) compared to Silymarin suspension; # compared to Silymarin gel.

The permeability of Silymarin across excised rat skin from produced Silymarin formulations was investigated as an indication to the expected in vivo behavior and was compared to the Silymarin suspension. The data in Figure 7 exhibited a significant enhancement (*p* < 0.05) in the amount of Silymarin permeated from Silymarin loaded transfersomal gel compared with the other formulations. The cumulative amounts of drug permeated through the skin from Silymarin transfersomal gel was 556.66 ± 23.92 µg/cm^2^, compared to 253.86 ± 26.48 µg/cm^2^ and 362.39 ± 22.18 µg/cm^2^ for the Silymarin suspension and Silymarin gel, respectively. Based on these findings, transfersomal formulation significantly (*p* < 0.05) improved Silymarin skin permeability.

In addition, the steady state transdermal flux (J_ss_) of Silymarin penetrated from different Silymarin formulations was in the following order: transfersomal gel (92.41 µg/cm^2^·h) > Silymarin gel 60.44 µg/cm^2^·h) > Silymarin aqueous suspension (46.83 µg/cm^2^·h). It was evident that Silymarin loaded transfersomal gel had the highest transdermal flux, which was significantly higher than those of all formulations, *p* < 0.05. Furthermore, drug permeation from the Silymarin transfersomal gel was improved by 1.97 folds, which was noticeably greater than that from Silymarin gel (60.44 µg/cm^2^·h) with an ER value of 1.29 (*p* < 0.05). The enhancement of skin permeability might be related, on the one hand, to the influence of non-ionic surfactant on enhancing the membrane fluidity, which facilitates drug diffusion, as well as surfactant and skin lipid interaction [5], and, on the other hand, to the flexibility and ultra-deformable structure of the transfersomal vesicles, which permit them to enter the stratum corneum and split through the lipid barrier [36].

### 3.7. Hypoglycemic Effect of the Developed Silymarin Formulations

**Figure 8:**
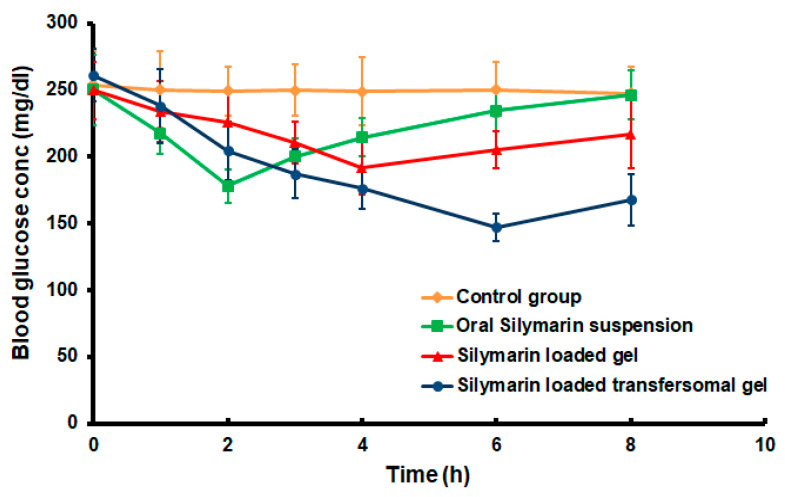
Blood glucose concentration after administration of different Silymarin formulations. Data represented as mean ± SD (*n* = 5).

**Figure 9:**
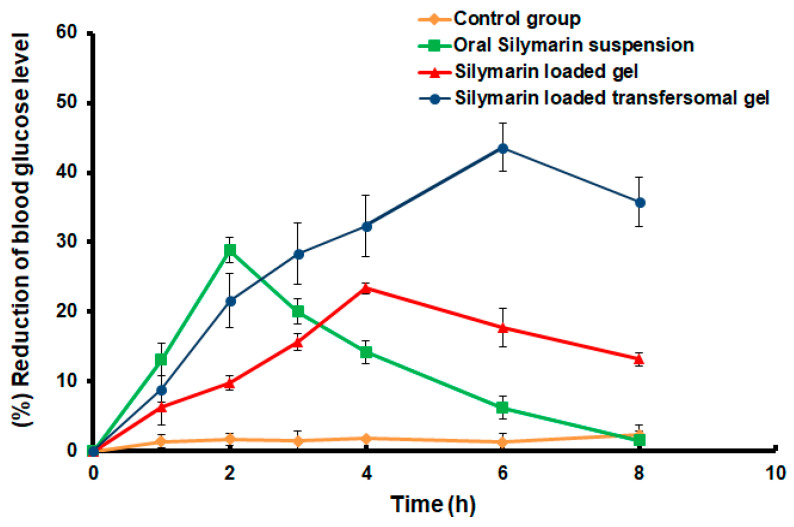
Percentage of blood glucose level reduction after administration of different Silymarin formulations. Data represented as mean ± SD (*n* = 5).

As shown in Figure 8 and Figure 9, the oral administration of the Silymarin suspension led to a significant and rapid reduction in blood glucose levels (*p* < 0.05), compared to the control untreated animals. The maximum reduction in blood glucose level (28.84 ± 1.82% was monitored after 2 h (T_max_) from the beginning of the experiment. However, Group five, transdermally medicated with the Silymarin-loaded transfersomal gel, demonstrated a delayed decline in blood glucose levels, with the maximum reduction percentage (T_max_) occurring after 6 h. The maximum hypoglycemic effect was about 43.61 ± 3.43%. In comparison to the oral application of the drug suspension, a shift in the T_max_ value toward the higher value for transdermal treatment demonstrated the controlled release behavior of the transfersomal gel formulation. The transdermal delivery of transfersomal gel loaded with Silymarin extended the hypoglycemic activity to 8 h, compared to orally administered Silymarin aqueous suspension, which ended after 6 h. Compared to the oral administration of Silymarin suspension, the delivery of Silymarin loaded transfersomal gel resulted in a prolonged reduction in blood glucose concentration. Our findings are consistent with those of Prasad et al. who reported that a transdermal formulation increased the bioavailability of pioglitazone by 2.26 times when compared to an oral administration of the drug [37].

Moreover, transfersomal gel had a considerably stronger hypoglycemic action (*p* < 0.05), compared to HPMC gel loaded with Silymarin. Figure 9 shows that the administration of transfersomal Silymarin gel resulted in a 43.61 ± 3.43 percent reduction in the blood glucose concentration after 6 h, whereas the application of HPMC gel resulted in a 23.38 ± 0.74 percent inhibition in blood glucose concentration after 4 h. The obtained results revealed a well correlation with the ex vivo permeation studies as the transfersomal gel exhibited a significant (*p* < 0.05) drug permeation difference in comparison to the HPMC gel after 6 h. These findings show that a sufficient amount of Silymarin was passed through the skin of rats to elicit a hypoglycemic activity, implying that transfersomes improved drug delivery through the skin and, hence, improved the therapeutic impact.

Moreover, the area above the curve (Figure 8) over 8 h (AAC_0–8h_) following the transdermal administration of transfersomal gel was determined to be (511.16 mg·h/dL), which was 1.77 times greater than the HPMC gel transdermal administration (288.32 mg·h/dL) and 2.12-fold higher than the oral administration of the SM suspension (240.67 mg·h/dL). Statistical analysis of the previous results demonstrated that transdermal administration of the optimized transfersomal gel loaded with Silymarin resulted in a significant increase in values of AAC_0–8h_, the percentage of the blood glucose reduction level, and T_max_ (*p* < 0.05).

## 4. Conclusions

In the current study, Silymarin-loaded transfersomes were prepared, optimized and assessed for their hypoglycemic effectiveness. The prepared vesicles were in the nano-size range and showing reasonable entrapment efficiency. Ex vivo skin permeation studies through abdominal full-thickness rabbit skins revealed a 2.2- and 1.5-fold increase in drug transdermal flux from transfersomal gel formulation compared to either the Silymarin suspension or Silymarin-loaded gel, respectively. Most importantly, the formulated Silymarin-loaded transfersomal gel elicited promising hypoglycemic efficacy in alloxan-induced diabetic rats, as manifested by a higher area above the blood glucose curve (AAC_0–8h_), compared to either the Silymarin-loaded HPMC gel or the orally administered Silymarin suspension. To sum up, encapsulation of Silymarin within transfersomal vesicles efficiently improved drug skin-permeation and, thereby, enhanced the hypoglycemic efficacy of the entrapped drug. Consequently, transfersomal drug delivery systems might represent potential carriers for circumventing the barrier function of skin and improving drug delivery via skin.

## Figures and Tables

**Table 1:** Variables of Box–Behnken design for Silymarin transfersomes formulations showing independent variables and their level of variation.

**Independent Variable**	**Character**	**Level of Variation**		
		**−1**	**0**	**+1**
Phospholipid concentration (mg)	X_1_	100	250	400
Surfactant concentration (mg)	X_2_	10	30	50
Sonication time (min)	X_3_	20	25	30
Dependent Responses				
(Y_1_) = Encapsulation efficiency EE%				
(Y_2_) = In vitro release of the drug after 6 h				

**Table 2:** The independent variables used for optimizing different transfersomal formulations and the detected results of dependent variables.

Formulation	Independent Variables			Dependent Variables	
	X_1_ (mg)	X_2_ (mg)	X_3_ (min)	Y_1_ (%)	Y_2_ (%)
F1	100	30	30	43.27 ± 1.22	43.03 ± 1.09
F2	250	30	25	66.80 ± 1.21	51.43 ± 1.13
F3	400	10	25	58.13 ± 0.32	51.81 ± 1.22
F4	100	50	25	39.80 ± 0.82	36.13 ± 0.70
F5	250	10	30	50.03 ± 1.05	51.97 ± 0.96
F6	250	30	25	67.50 ± 1.32	52.02 ± 0.66
F7	250	30	25	66.58 ± 1.51	51.50 ± 0.77
F8	250	50	20	55.03 ± 1.05	49.96 ± 0.87
F9	400	30	20	70.13 ± 0.80	58.13 ± 1.56
F10	100	30	20	47.87 ± 1.31	38.03 ± 1.56
F11	400	30	30	63.93 ± 0.90	60.01 ± 0.59
F12	400	50	25	56.17 ± 0.76	50.03 ± 1.13
F13	250	50	30	53.10 ± 0.56	47.96 ± 0.78
F14	250	10	20	57.17 ± 0.47	44.91 ± 0.82
F15	100	10	25	33.10 ± 0.66	28.35 ± 0.28

X_1_—amount of phospholipid (mg); X_2_—amount of surfactant (mg); X_3_—sonication time (min); Y_1_—EE (%); Y_2_—In vitro release (%).

**Table 3:** Predicted and observed results of the optimized Silymarin loaded transfersomes formulation.

**Independent Variables**	**Symbol**	**Goal**
Phospholipid concentration (mg)	X_1_	In range
Surfactant concentration (mg)	X_2_	In range
Sonication time (min)	X_3_	In range
Dependent variables	Predicted values	Observed values
R_1_ (%)	70.13 ± 2.62	68.61 ± 2.36
R_2_ (%)	58.33 ± 1.92	57.33 ± 2.07

**Table 4:** Results of statistical analysis of all dependent variables Y_1_ and Y_2_.

**Source**	**Y_1_**		**Y_2_**	
	***F*-Value**	***p*-Value**	***F*-Value**	***p*-Value**
Model	317.13	<0.0001 *	146.99	<0.0001 *
X_1_—Phospholipid (mg)	1511.95	<0.0001 *	934.30	<0.0001 *
X_2_—Surfactant (mg)	6.84	0.0474 *	8.36	0.0342 *
X_3_—Sonication time (min)	83.96	0.0003 *	24.04	0.0045 *
Lack of Fit	3.58	0.2261	11.23	0.0829
R^2^ analysis				
R²	0.9983		0.9962	
Adjusted R²	0.9951		0.9895	
Predicted R²	0.9758		0.9427	
Adequate Precision	58.0476		43.8283	

X_1_—Phospholipid concentration (mg); X_2_—Surfactant concentration (mg); X_3_—Sonication time (min); Y_1_—EE (%); Y_2_—In vitro release (%); * significant.

**Table 5:** Characterization of gel and transfersomal gel formulations encapsulating Silymarin.

Properties	Silymarin Gel	Silymarin Transfersomal Gel
Visual inspection	Smooth and homogenous	Smooth and homogenous
pH	6.89 ± 0.31	7.05 ± 0.45
Spreadability (mm)	52.9 ± 2.4	55.35 ± 3.03 *
Viscosity (Pa)	5.96 ± 0.77	6.27 ± 0.63 Pa *
Drug content (%)	99.13 ± 0.42	99.35 ± 0.61

Values are stated as mean ± (SD), * *p* < 0.05 compared to Silymarin gel.

## Data Availability

Not applicable.

## Supplementary Materials

The following supporting information can be downloaded at: https://www.mdpi.com/article/10.3390/polym14030508/s1, Figure S1: Schematic diagram of locally fabricated diffusion cell.
