# Supplementary material for: Experimental Design and Optimization of Nano-Transfersomal Gel to Enhance the Hypoglycemic Activity of Silymarin
Source: Polymers (Basel). 2022 Jan 27;14(3):508. doi: 10.3390/polym14030508 (PMC8838802; doi:10.3390/polym14030508)
Supplement: Supplementary file 1 [file polymers-14-00508-s001.zip › polymers-1563806-supplementary.pdf]

Experimental Design and Optimization of Nano-Transfersomal Gel to Enhance the Hypoglycemic Activity of Silymarin

Marwa H. Abdallah [1,2,*], Amr S. Abu Lila [2], Seham Mohammed Shawky [3], Khaled Almansour [1], Farhan Alshammari [1], El-Sayed Khafagy [4,5] and Tarek Saad Makram [6]

1 Department of Pharmaceutics, College of Pharmacy, University of Ha'il, Ha'il 81442, Saudi Arabia;
kh.almansour@uoh.edu.sa (K.A.); frh.alshammari@uoh.edu.sa (F.A.)
2 Department of Pharmaceutics and Industrial Pharmacy, Faculty of Pharmacy, Zagazig University,
Zagazig 44519 , Egypt; a.abulila@uoh.edu.sa (A.S.A)
3 Department of Pharmaceutics and Pharmaceutical Technology, Faculty of Pharmacy, Al-Azhar University, Cairo 11651, Egypt; sehamshawky@azhar.edu.eg (S.M.S)
4 Department of Pharmaceutics, College of Pharmacy, Prince Sattam Bin Abdulaziz University,
Al-Kharj 11942, Saudi Arabia; e.khafagy@psau.edu.sa (E.-S.K)
5 Department of Pharmaceutics and Industrial Pharmacy, Faculty of Pharmacy, Suez Canal University,
Ismailia 41552, Egypt
6 Department of Pharmaceutics and Industrial Pharmacy, Faculty of Pharmacy, October 6 University,
Al Mehwar Al Markazi 12511, Egypt; tareksaadmakram@yahoo.com (T.S.M)

* Correspondence: mh.abdallah@uoh.edu.sa (M.H.A)

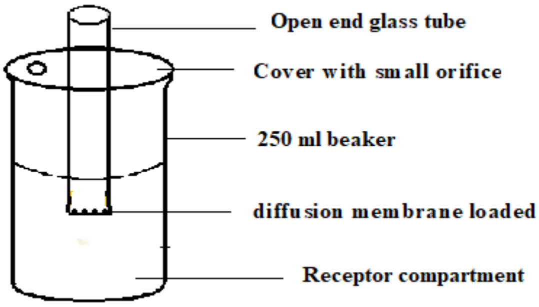

Figure S1. Schematic diagram of locally fabricated diffusion cell.
